# Metabolic engineering of *Pichia pastoris* for *myo*-inositol production by dynamic regulation of central metabolism

**DOI:** 10.1186/s12934-022-01837-x

**Published:** 2022-06-03

**Authors:** Qiquan Zhang, Xiaolu Wang, Huiying Luo, Yaru Wang, Yuan Wang, Tao Tu, Xing Qin, Xiaoyun Su, Huoqing Huang, Bin Yao, Yingguo Bai, Jie Zhang

**Affiliations:** State Key Laboratory of Animal Nutrition, Institute of Animal Science, Chinese Academy of Agricultural Sciences, No.2 Yuanmingyuan West Road, Haidian district, Beijing, 100193 China

**Keywords:** *Myo*-inositol, *Pichia pastoris*, Metabolic engineering, Dynamic regulation, High-cell-density fermentation

## Abstract

**Background:**

The methylotrophic budding yeast *Pichia pastoris* GS115 is a powerful expression system and hundreds of heterologous proteins have been successfully expressed in this strain. Recently, *P. pastoris* has also been exploited as an attractive cell factory for the production of high-value biochemicals due to Generally Recognized as Safe (GRAS) status and high growth rate of this yeast strain. However, appropriate regulation of metabolic flux distribution between cell growth and product biosynthesis is still a cumbersome task for achieving efficient biochemical production.

**Results:**

In this study, *P. pastoris* was exploited for high inositol production using an effective dynamic regulation strategy. Through enhancing native inositol biosynthesis pathway, knocking out inositol transporters, and slowing down carbon flux of glycolysis, an inositol-producing mutant was successfully developed and low inositol production of 0.71 g/L was obtained. The inositol production was further improved by 12.7% through introduction of heterologous inositol-3-phosphate synthase (IPS) and inositol monophosphatase (IMP) which catalyzed the rate-limiting steps for inositol biosynthesis. To control metabolic flux distribution between cell growth and inositol production, the promoters of glucose-6-phosphate dehydrogenase (ZWF), glucose-6-phosphate isomerase (PGI) and 6-phosphofructokinase (PFK1) genes were replaced with a glycerol inducible promoter. Consequently, the mutant strain could be switched from growth mode to production mode by supplementing glycerol and glucose sequentially, leading to an increase of about 4.9-fold in inositol formation. Ultimately, the dissolved oxygen condition in high-cell-density fermentation was optimized, resulting in a high production of 30.71 g/L inositol (~ 40-fold higher than the baseline strain).

**Conclusions:**

The GRAS *P. pastoris* was engineered as an efficient inositol producer for the first time. Dynamic regulation of cell growth and inositol production was achieved via substrate-dependent modulation of glycolysis and pentose phosphate pathways and the highest inositol titer reported to date by a yeast cell factory was obtained. Results from this study provide valuable guidance for engineering of *P. pastoris* for the production of other high-value bioproducts.

**Supplementary Information:**

The online version contains supplementary material available at 10.1186/s12934-022-01837-x.

## Background

*Myo*-inositol (inositol) is a carbocyclic sugar widely distributed in plants, animals and microorganisms [1]. Inositol has nine possible structural isomers, which have been extensively characterized and studied [2, 3]. Although the requirements of inositol for monogastric animals and humans are generally met by their cellular biosynthesis, dietary inositol has been found to be beneficial for treating neurological and endocrine diseases such as depression, panic disorder, diabetes and insulin resistance [4, 5]. Recent studies also demonstrated that inositol and its derivatives have an anti-aging effect on mammals including humans [6, 7]. For most of the aquatic animals, inositol is regarded as an essential nutrient and its deficiency could lead to decreased growth rate, fin erosion, dark skin colouration, and fatty liver disease [8–10]. Therefore, inositol is broadly applied as aquaculture feed supplement. In addition, due to its positive effect on lipid metabolism, bone formation and skeletal muscle metabolism [11, 12], inositol is also used as an active supplement in functional food and cosmetics industries.

The production of inositol can be achieved by phytate hydrolysis, in vitro enzymatic synthesis, and in vivo microbial biosynthesis [13, 14]. At present, inositol is mainly produced by phytate hydrolysis under harsh conditions (low pH, high temperature and pressure) which is unfriendly to the environment. Recently, in vitro enzymatic synthesis routes were proposed for inositol production from glucose or starch [15, 16]. However, the enzymatic production of inositol has suffered from a number of draw-backs, such as complex product separation process and instability of the catalytic enzymes, making this route economically uncompetitive. In vivo microbial biosynthesis of inositol is considered as the most promising alternative to the conventional phytate hydrolysis method owing to its low production cost and environmental-friendly production process. So far, *Escherichia coli* is the main host studied for microbial production of inositol though metabolic engineering [14, 17, 18]. By blocking or reducing the carbon fluxes toward glycolysis and pentose phosphate pathways, the inositol production was significantly increased [14, 18]. However, one of the main disadvantages of using *E. coli* strain for commercial inositol production is the safety concerns associated with the lack of the GRAS (Generally Recognized as Safe) status of this bacteria.

*Pichia pastoris* (recently classified as *Komagataella phaffii*), is a methylotrophic budding yeast and could grow rapidly on defined chemical medium with glucose or glycerol as substrate [19]. *P. pastoris* has been extensively used as an efficient platform for heterologous recombinant proteins production due to its GRAS status, rapid growth rate and ability for high-cell-density fermentation [20]. Recently, *P. pastoris* was also exploited as a robust host for value-added biochemicals production, including S-adenosyl-L-methionine (SAM) [21], malic acid [22], lycopene [23], etc. indicating this yeast strain is indeed a versatile microbial cell factory.

**Fig. 1:**
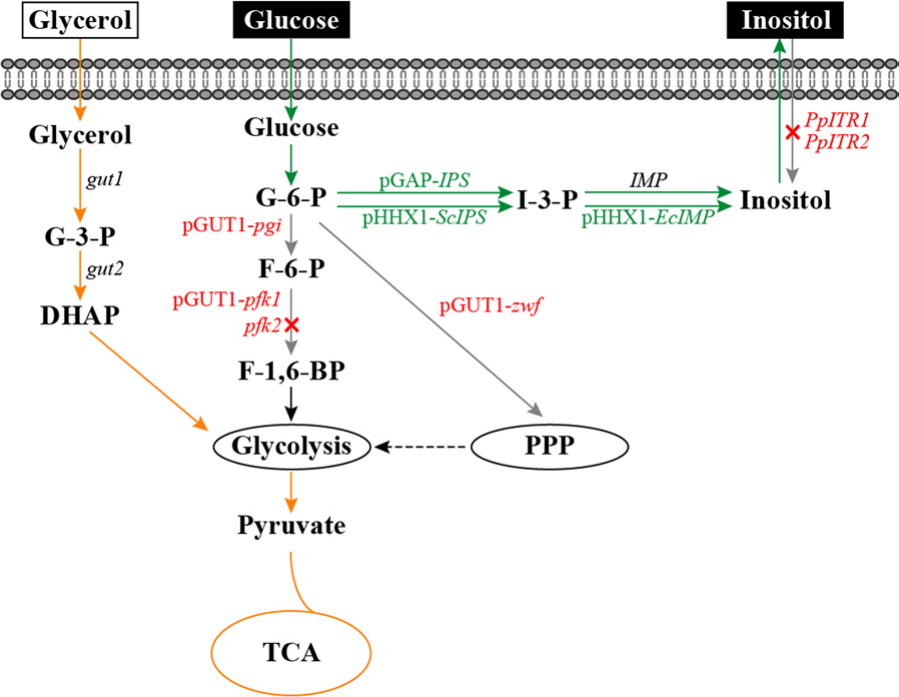
Strategies for high-titer production of inositol in *P. pastoris*. Green arrows indicate the inositol biosynthesis pathway; orange arrows indicate glycerol catabolic pathway; genes in green indicate overexpression; genes in red indicate deletion or attenuation. G-3-P, glycerol-3-phosphate; DHAP, dihidroxyacetone-phosphate; G-6-P, glucose-6-phosphate; F-6-P, fructose-6-phosphate; F-1,6-BP, fructose-1,6-bisphosphate; I-3-P, inositol-3-phosphate; *gut1*, glycerol kinase; *gut2*, FAD-dependent glycerol-3-phosphate dehydrogenase; *pgi*, glucose-6-phosphate isomerase; *pfk1*, α-subunit of heterooctameric phosphofructokinase; *pfk2*, β-subunit of heterooctameric phosphofructokinase; *IPS*, inositol-3-phosphate synthase; *IMP*, inositol monophosphatase; *zwf*, glucose-6-phosphate dehydrogenase; *PpITR1*/*PpITR2*, inositol transporters

In this study, we aimed to engineer *P. pastoris* for efficient inositol production by dynamic regulation of carbon flux distribution (Fig. 1). First, an inositol-producing strain was constructed by enhancing the expression of endogenous *IPS* (encoding inositol-3-phosphate synthase, PAS_chr2-2_0113) gene which is the rate-limiting step for inositol biosynthesis and knocking out the *PpITR1* (PAS_chr2-1_0489)/*PpITR2* (PAS_chr4_0828) and *pfk2* (PAS_chr1-4_0047) genes which are inositol transporters and β-subunit of 6-phosphofructokinase gene, respectively. Second, two heterologous enzymes IPS and IMP (inositol monophosphatase, PAS_chr4_0730) were overexpressed to further increase the inositol production. Third, the promoters of *zwf* (encoding glucose-6-phosphate dehydrogenase), *pgi* (encoding glucose-6-phosphate isomerase), and *pfk1* (encoding α-subunit of 6-phosphofructokinase, PAS_chr2-1_0402) genes were replaced with the promoter of *gut1* (encoding glycerol kinase, PAS_chr4_0783) gene. Consequently, the carbon flux distribution could be regulated in the presence of glucose via carbon catabolite repression (CCR). Last, the effect of oxygen availability on inositol production was investigated. The obtained mutant produced 30.71 g/L inositol in a high-cell-density fermentation in the 10 L bioreactor. Results from this study provide valuable guidance and promising strategies for engineering other yeast strains for inositol production.

## Results

### Metabolic engineering of the native inositol biosynthesis pathway and transport system in *P. pastoris*

**Fig. 2:**
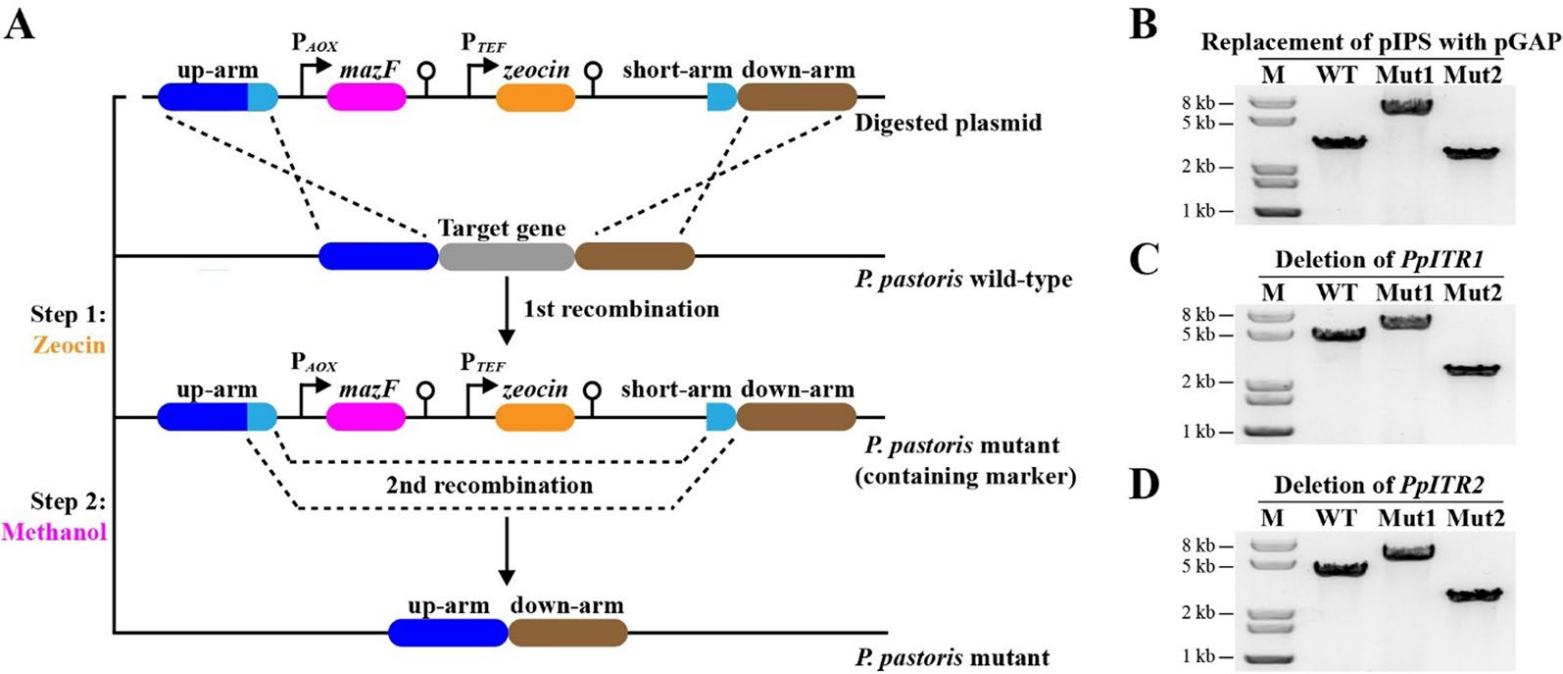
Markerless genome editing in *P. pastoris* using *mazF*-*zeoR* counter-selection approach. **A** Schematic illustrating the work flow of markerless genome editing system using *mazF*-*zeoR* counter-selection approach. Three homology arms with different lengths (up-arm and down-arm, ~ 1000–1200 bp; short-arm, ~ 100–300 bp) were used for gene deletion or integration. Two screening steps including two rounds of homologous recombination are involved. In step 1, the digested plasmid was transformed into *P. pastoris* under the selection of antibiotic zeocin, allowing the integration of “*mazF*-*zeocin*-short-arm” fragment onto the chromosome and the elimination of the target gene. In step 2, the obtained transformants were cultivated in BMMY medium containing methanol as the sole carbon source to induce the expression of *mazF* gene. The MazF selection stress promotes the removal of “*mazF*-*zeocin*” cassette through the second round of homologous recombination. **B** PCR analysis of JQ01 mutant after each selection step. **C, D** PCR analysis of JQ02 mutant after each selection step. M, 1-kb DNA ladder; WT, genomic DNA of *P. pastoris* GS115 as the control; Mut1, mutant strain after first round of recombination; Mut2, mutant strain after second round of recombination

A markerless genome editing tool was initially developed based on *mazF*-*zeoR* counter-selection approach with some modifications [24]. Previous study demonstrated that the *mazF*-*zeoR* counter-selection approach is a highly efficient genome editing tool for *P. pastoris*. However, an unwanted scar (CYC1TT) was left in the genome after each round of gene editing (Additional file 1: Fig. S1) [24], which would limit the possible precise genomic modifications and increase the risk of homologous recombination between the scar sequences after multiple rounds of editing. In this study, the two CYC1TT sequences were removed, and a short-arm sequence (~ 100–300 bp) homologous to the 3’-end sequence of up-arm was inserted. Consequently, the up-arm and down-arm (~ 1000–1200 bp/each) which were much longer than short-arm would dominate the first round of recombination, while the short-arm would control the second round of recombination (Fig. 2A). Using the modified *mazF*-*zeoR* counter-selection approach, markerless genome editing could be achieved after two rounds of homologous recombination.

**Fig. 3:**
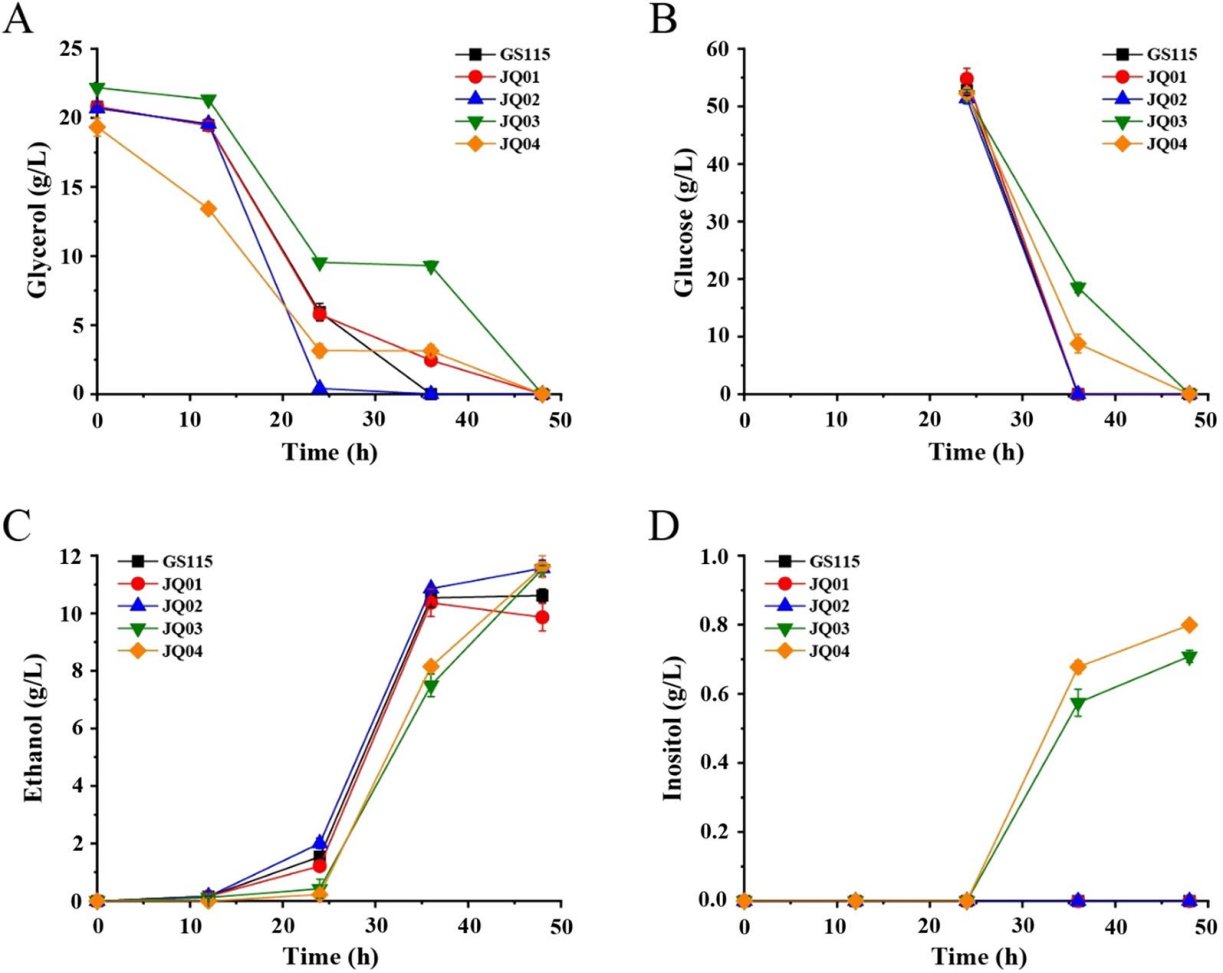
Fed-batch fermentation profiles of *P. pastoris* GS115, JQ01, JQ02, JQ03, and JQ04. Strains were fermented in inorganic salt medium containing 20 g/L glycerol, thereafter 50 g/L glucose was added into the medium at the 24 h of fermentation for inositol production. **A** Glycerol consumption. **B** Glucose consumption. **C** Ethanol production. **D** Inositol production

The biosynthesis of inositol from the precursor glucose-6-phosphate (G-6-P) involves two crucial enzymes, IPS which can convert G-6-P to inositol-3-phosphate (I-3-P) and IMP which catalyzes the formation of inositol from I-3-P. Different from *E. coli*, *P. pastoris* has a native biosynthetic pathway for inositol production. However, the inositol biosynthesis in yeast is strictly regulated mainly by controlling the transcription of *IPS* gene [25]. Therefore, the promoter of *IPS* gene was replaced with the strong constitutive promoter pGAP (the promoter of glyceraldehyde-3-phosphate dehydrogenase gene) using the newly established *mazF*-*zeoR* counter-selection approach (Fig. 2B), creating mutant strain JQ01. It is reported that two transporters (ITR1 and ITR2) are responsible for extracellular inositol uptake in *Saccharomyces cerevisiae* [26]. By analyzing the genomic sequence of *P. pastoris*, two possible inositol transporters, PpITR1 and PpITR2, were identified to have strong similarity to ITR1 and ITR2 of *S. cerevisiae*, respectively. In order to decrease the potential inositol reassimilation and increase the inositol production, the two inositol transporter genes were deleted in strain JQ01 (Fig. 2C, D), generating JQ02. Fermentation results showed that ethanol was the major product for JQ01 and JQ02 (Fig. 3C). However, no detectable inositol production was observed in the fermentation broth of JQ01 and JQ02 (Fig. 3D), suggesting that some other factors might affect the production of inositol in *P. pastoris*.

### Inositol production by slowing down the carbon flux of glycolytic pathway

The precursor for inositol biosynthesis is G-6-P, which is also an important intermediate for glycolysis pathway. Although the expression of *IPS* gene was enhanced, G-6-P might be still catabolized mainly in glycolysis pathway in *P. pastoris*, leading to negligible inositol production. In order to increase the G-6-P availability for inositol biosynthesis, the key gene—*pgi*—involved in catalyzing the conversion of G-6-P to fructose-6-phosphate (F-6-P) in glycolysis was initially selected to be deleted (Fig. 1). However, the attempt was unsuccessful using our *mazF*-*zeoR* counter-selection approach (data not shown), suggesting that the *pgi* gene plays a vital role in the central carbon metabolism of *P. pastoris*. Then, the *pfk2* gene which is the β-subunit of heterooctameric phosphofructokinase in *P. pastoris* was chosen as another target gene to be deleted (Fig. 1). Fortunately, *pfk2* gene was successfully deleted in strain JQ02, yielding mutant JQ03. Shake-flask fermentations were then carried out with wild-type strain and JQ03 to test their inositol production, using glycerol (initial carbon source for cell growth) and glucose (supplemented at 24 h of fermentation) as substrates. Results showed that the ethanol productions of wild-type strain and JQ03 mutant reached 10.61 and 11.54 g/L, respectively, indicating that ethanol was still the major product of these two strains (Fig. 3C). As expected, there was no detectable inositol production for wild-type strain. However, 0.71 g/L inositol was produced by mutant JQ03 at the end of fermentation (Fig. 3D). The ethanol production of mutant JQ03 was detected throughout the fermentation process, whereas the inositol accumulation was only observed after the addition of glucose (Fig. 3A–D). These results demonstrated that slowing down the carbon flux of the glycolytic pathway was an effective strategy to increase the G-6-P supply for inositol production.

### **Enhancement of inositol production by introduction of heterologous*ScIPS*and*EcIMP***

High inositol production was achieved in *E. coli* when plasmid was used for *IPS* and *IMP* overexpression [14, 18], which would usually give higher expression than single chromosome integration. Therefore, iterative integration of multiple-copy of *IPS* and *IMP* genes in *P. pastoris* was conducted in order to further enhance the inositol production. The *ScIPS* from *S. cerevisiae* and the *EcIMP* from *E. coli* which have been found to be efficient for inositol biosynthesis were selected and integrated into the *PpITR1* locus of JQ03, generating mutant JQ04. Bidirectional promoter pHHX1 was employed for *ScIPS* and *EcIMP* overexpression [27]. Shake-flask fermentation showed that mutant JQ04 produced 0.80 g/L inositol, which was just slightly increased (12.7%) compared to that of strain JQ03 (Fig. 3D). In addition, the ethanol production of JQ04 reached 11.62 g/L, which was kept at the similar level to that of JQ03 (Fig. 3C). These results demonstrated that overexpression of heterologous *ScIPS* and *EcIMP* only had limited effect on inositol biosynthesis, implying that the supply of precursor G-6-P might be still not sufficient for inositol production in JQ04 and need to be further improved.

### Further improvement of inositol production by dynamic regulation of glycolysis and pentose phosphate pathways

As demonstrated above, when glucose was used as carbon source for inositol production in *P. pastoris*, the carbon flux was mainly driven towards cell growth, primarily glycolysis and pentose phosphate pathways. In order to enhance the inositol production, these two pathways should be blocked or weakened. However, totally block the glycolysis and pentose phosphate pathways could lead to severe inhibition of cell growth [28]. Dynamic regulation is considered to be an effective strategy to modulate the balance between cell growth and product formation [29, 30]. Here, this strategy was applied to enhance the inositol production from glucose in *P. pastoris*. It is known that glycerol is an efficient carbon source for *P. pastoris*, but its utilization is repressed in presence of glucose. This repression is usually achieved by regulating the expression of *gut1* gene which encodes glycerol kinase and mediates the first step of glycerol utilization in *P. pastoris*. In this study, we attempted to use the promoter of *gut1* gene (pGUT1) to control the carbon flux of glycolysis and pentose phosphate pathways through carbon catabolite repression (CCR). Therefore, the promoters of *zwf*, *pgi* and *pfk1* genes were replaced with the glycerol-inducible promoter pGUT1 in JQ04 (Fig. 1). The resultant strains were named as JQ05 (JQ04::pGUT1-*zwf*), JQ06 (JQ04::pGUT1-*pgi*), JQ07 (JQ04::pGUT1-*zwf*::pGUT1-*pgi*) and JQ08 (JQ07::pGUT1-*pfk1*). Consequently, the cell growth would not be disturbed when glycerol was used as carbon source, while the expressions of *zwf*, *pgi* and *pfk1* genes could be inhibited through the addition of glucose, which could be beneficial for inositol production.

**Fig. 4:**
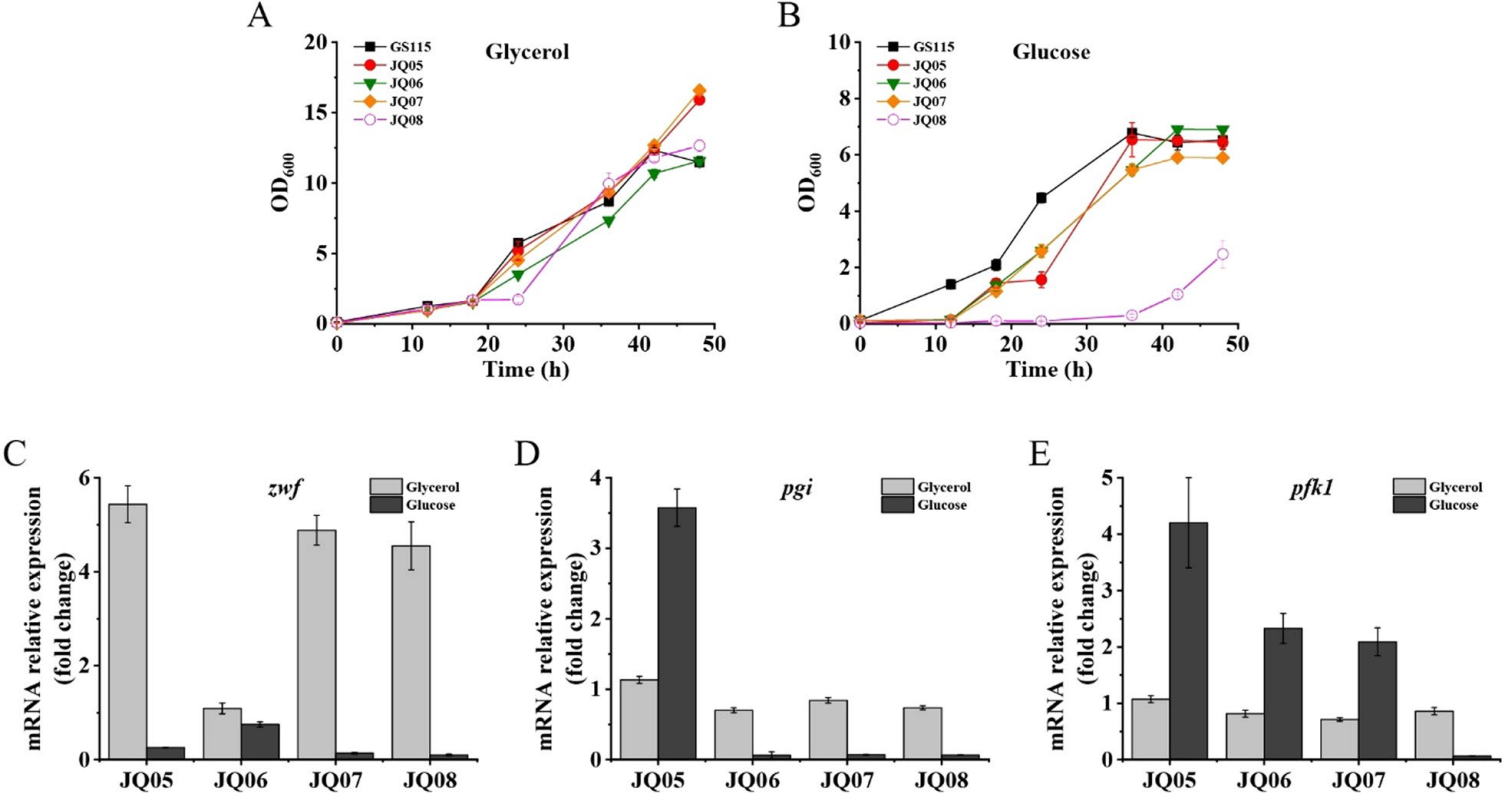
Growth profiles and *zwf*, *pgi*, and *pfk1* expressions of *P. pastoris* GS115, JQ05, JQ06, JQ07, and JQ08 grown in inorganic salt medium with glucose or glycerol as sole carbon source. **A** Growth profiles of various strains cultivated in glycerol medium. **B** Growth profiles of various strains cultivated in glucose medium. **C** qPCR results showing the mRNA expressions of *zwf* gene of various strains. **D** qPCR results showing the mRNA expressions of *pgi* gene of various strains. **E** qPCR results showing the mRNA expressions of *pfk1* gene of various strains

Mutants JQ05, JQ06, JQ07 and JQ08 were then cultured in inorganic salt medium with 20 g/L glucose or glycerol as sole carbon source to characterize their growth profiles. *P. pastoris* GS115 wild-type strain was used as the control. Results showed that all tested strains grew well in the glycerol medium, whereas prolonged lag-phase was observed for mutants JQ05, JQ06, JQ07 and JQ08 cultivated in glucose medium compared to wild-type strain (Fig. 4A, B), which were largely consistent with our expectations. A 12 h lag-phase was observed for strain JQ05, JQ06 and JQ07 (Fig. 4B). However, the lag-phase of mutant JQ08 was extended to 36 h, suggesting that the glycolysis and pentose phosphate pathways of JQ08 were strictly controlled.

In order to further verify the expressions of *zwf*, *pgi* and *pfk1* genes in mutants JQ05, JQ06, JQ07 and JQ08, RT-qPCR was performed. The qPCR results showed that the expression of *zwf* gene in strains JQ05, JQ07 and JQ08 cultured in glucose medium were significantly decreased compared to those of JQ05, JQ07 and JQ08 cultured in glycerol medium (Fig. 4C), due to the glucose-mediated repression on pGUT1. In contrast, the expression of *zwf* gene in strain JQ06 possessing its native *zwf* promoter was kept at the similar level whether the carbon source in the medium was glucose or glycerol (Fig. 4C). As for *pgi* and *pfk1* expressions, the native promoters of *pgi* (JQ05) and *pfk1* (JQ05, JQ06 and JQ07) gave a higher transcription activity in glucose medium than those in glycerol medium (Fig. 4D, E). However, after the replacement of the native promoters of *pgi* and *pfk1* with glycerol-inducible promoter pGUT1, the expressions of *pgi* (JQ06, JQ07 and JQ08) and *pfk1* (JQ08) were greatly reduced in glucose medium compared to those in glycerol medium (Fig. 4D, E). These results are coincident with the results that the growth of mutants JQ05, JQ06, JQ07 and JQ08 were inhibited in the presence of glucose (Fig. 4B), demonstrating that the expressions of *zwf*, *pgi* and *pfk1* genes in these mutants could be regulated by using glucose or glycerol as carbon source.

**Fig. 5:**
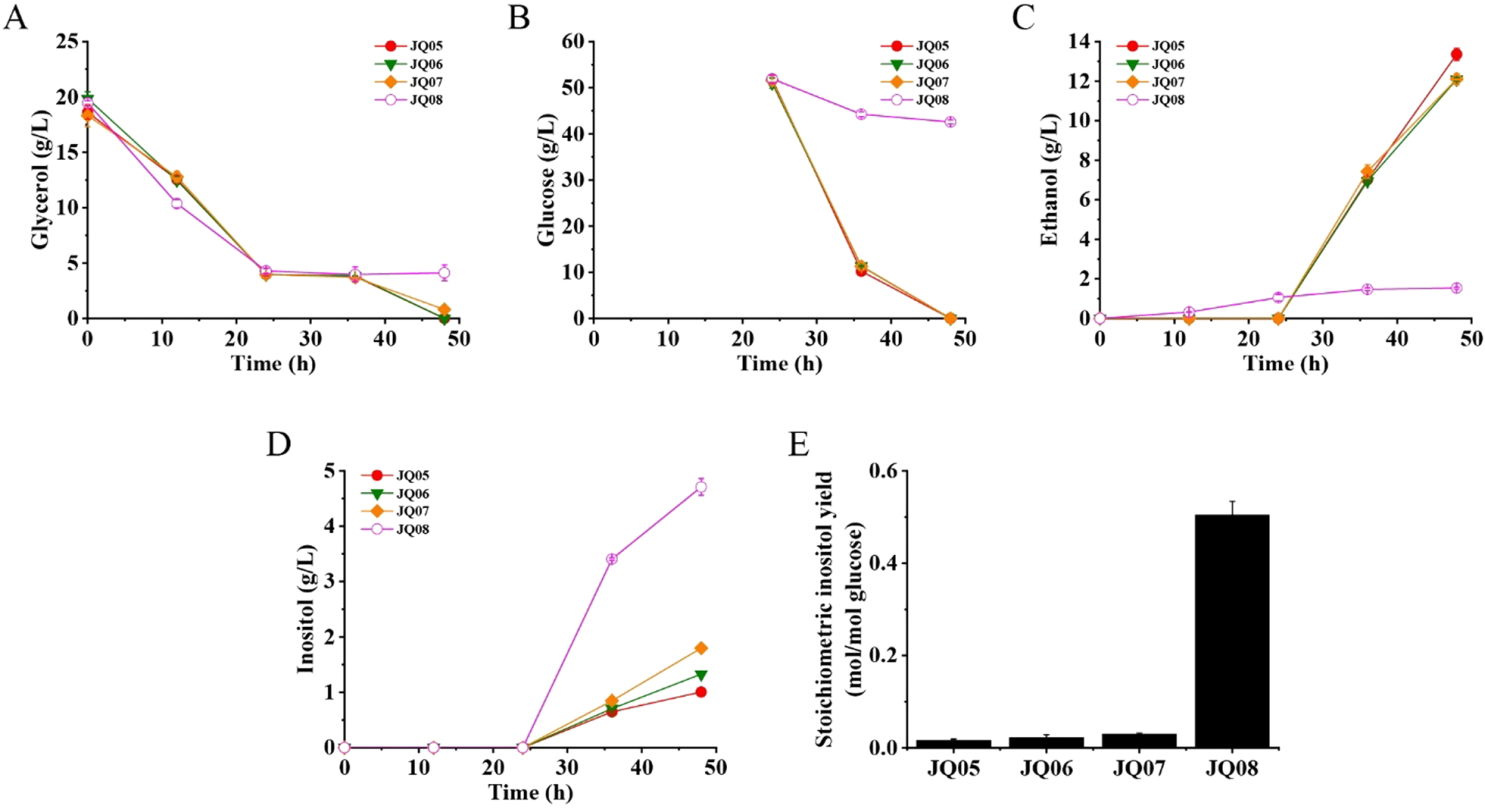
Fed-batch fermentation profiles of mutants JQ05, JQ06, JQ07, and JQ08. All mutants were fermented in inorganic salt medium containing 20 g/L glycerol. At the 24 h of fermentation, 50 g/L glucose was added into the medium for inositol production. **A** Glycerol consumption. **B** Glucose consumption. **C** Ethanol production. **D** Inositol production. **E** Stoichiometric yield of inositol

Shake-flask fermentations with mutants JQ05, JQ06, JQ07 and JQ08 were then performed using glycerol (for cell growth) and glucose (for inositol production) as substrates. Fermentation results showed that 1.00, 1.33 and 1.80 g/L inositol were produced by mutants JQ05, JQ06 and JQ07, respectively, which were increased by 25.0%, 66.3% and 125%, respectively, compared to that of mutant JQ04 (Fig. 5D). The ethanol productions of mutants JQ05, JQ06 and JQ07 were 13.35, 12.09 and 12.11 g/L, respectively, which were still much higher than inositol productions in these strains (Fig. 5C). The glycerol and glucose consumption profiles of JQ05, JQ06 and JQ07 were similar with those of JQ04 (Fig. 5A, B). In contrast, the carbon source consumption of mutant JQ08, especially the glucose consumption, was severely restrained (Fig. 5B), due to the block of glycolysis and pentose phosphate pathways. The ethanol production of JQ08 was reduced to 1.54 g/L (Fig. 5C). However, the inositol production was significantly enhanced in JQ08 (Fig. 5D). As high as 4.71 g/L inositol was generated in JQ08 at the end of fermentation, representing an increase of about 4.9-fold compared to that of mutant JQ04. Total 9.35 g/L glucose was utilized in the fermentation process of JQ08, indicating that the inositol yield of JQ08 reached 0.50 mol/mol glucose (Fig. 5E). However, the inositol yields of JQ05, JQ06 and JQ07 were just 0.02, 0.02 and 0.03 mol/mol glucose, respectively. These results indicated that dynamic regulation of glycolysis and pentose phosphate pathways was an effective strategy to enhance the inositol production in *P. pastoris*.

### High-cell-density fermentation with strain JQ08

**Fig. 6:**
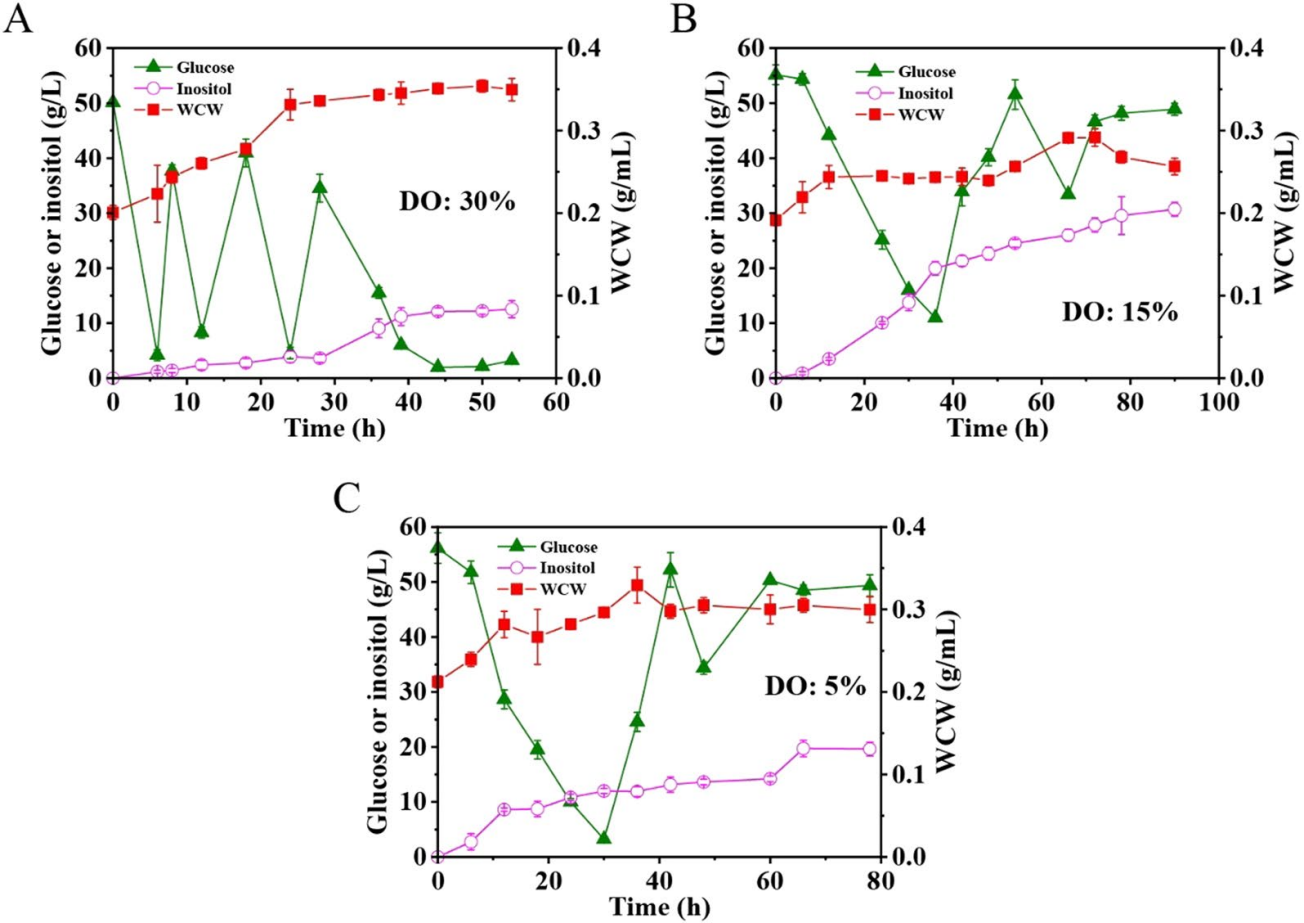
High-cell-density fermentation with mutant JQ08. The fermentations were performed in a 10 L bioreactor. Strain JQ08 was initial cultivated in the inorganic salt medium containing glycerol as the sole carbon source. When WCW reached about 0.2 g/mL, glucose was added into the medium for inositol production. Glucose was supplemented in batches. The DO was maintained at about 30% when glycerol was used as the carbon source for efficient cell growth. Then the level of DO was changed to 30% **A**, 15% **B** or 5% **C** after the addition of glucose for inositol biosynthesis

High inositol production and yield were obtained for strain JQ08 using glycerol and glucose as carbon sources. In order to comprehensively assess the fermentation capacity of JQ08, high-cell-density fermentation was carried out in a 10 L bioreactor. Glycerol was used as the initial carbon source for cell growth. When the wet cell weight (WCW) reached about 0.2 g/mL, glucose was then supplemented into the fermentation medium for inositol production. Initially, the level of dissolved oxygen (DO) was maintained at about 30% throughout the fermentation. The concentration of inositol reached 12.56 g/L at the end of fermentation (Fig. 6A). It is reported that oxygen limitation could strongly influence the core metabolism of *P. pastoris* by causing energy deprivation [31], and hypoxic condition has beneficial effect on recombinant protein secretion [32]. Therefore, lower DO levels were utilized to testify the effect of oxygen availability on inositol production in strain JQ08. Results showed that when DO levels were reduced to 15% and 5% after the addition of glucose, the inositol production of JQ08 reached 30.71 and 19.63 g/L (Fig. 6B, C), respectively, which were enhanced by 145% and 56.3%, respectively, compared to that of JQ08 fermented under the 30% DO condition. The results suggested that moderate control of oxygen availability has a positive effect on inositol production for mutant JQ08.

## Discussion

In recent years, inositol and its derivatives have received considerable attention for their commercial applications in the fields of medicine, functional food, cosmetics and feed [33, 34]. Traditionally, the inositol was primarily produced by hydrolyzing plant-derived phytate, which would result in severe environmental pollution. Microbial biosynthesis of inositol has been regarded as one of the most promising alternatives to the phytate hydrolysis method. Numerous studies have been reported for metabolic engineering of different strains for inositol production [35]. To date, *E. coli* is the most efficient host for inositol biosynthesis, and high inositol production and yield were achieved in this strain [14, 18]. However, biochemicals produced by *E. coli* is restricted for medicine and food use purpose. In addition, to enhance the expression of genes involved in inositol biosynthesis, multi-plasmid expression approach was used in the most current reports [14, 18]. This on one hand brings extra burden to cellular metabolism; on the other, losing plasmid during the fermentation would lead to undulation in production. In this work, the methylotrophic yeast *P. pastoris*, an effective heterologous protein expression system, was exploited for efficient inositol production by dynamic regulation of carbon flux distribution without using plasmid expression system.

The main challenge for high-level inositol biosynthesis using microbial cell factory is the sufficient supply of G-6-P, which is the precursor for inositol biosynthesis and the essential intermediate for glycolysis and pentose phosphate pathways. Disruption or attenuation of glycolysis and pentose phosphate pathways could increase the yield of bioproducts derived directly from G-6-P. This strategy has been employed to glucaric acid and inositol production [35, 36]. In this study, we initially wanted to construct an inositol producing strain by manipulating the native inositol biosynthesis pathway and transport system in *P. pastoris*. However, the inositol accumulation was not observed in mutant JQ02 (Fig. 3D), suggesting that G-6-P was mainly consumed in glycolysis and pentose phosphate pathways. Totally block the glycolysis and pentose phosphate pathways would be an optimal option to enhance the supply of G-6-P, whereas the attempts consistently failed in *P. pastoris*. Mutant JQ03 with attenuated glycolysis pathway was successfully constructed by deletion of *pfk2* gene, and inositol production was detected in the fermentation broth of JQ03 (Fig. 3D), which substantiated that slowing down the carbon flux of glycolytic pathway was beneficial for the production of inositol.

Appropriate modulation of the balance between cell growth and product formation is another challenge for inositol production from glucose. By using different carbon sources with direct access to growth and production modules, biochemical synthesis could be significantly improved [37, 38]. In this study, glycerol and glucose, which are both preferable carbon sources for *P. pastoris*, were selected and applied to support the cell growth and inositol production, respectively. In order to conserve more glucose for inositol formation, the promoters of key metabolic genes involved in glycolysis and pentose phosphate pathways, including *zwf*, *pgi* and *pfk1*, were replaced with the glycerol-inducible promoter pGUT1. Therefore, dynamic regulation of cell growth and inositol production was achieved via supplementing glycerol and glucose sequentially. The growth profile and RT-qPCR result of JQ08 substantiated that the expressions of *zwf*, *pgi* and *pfk1* were successfully regulated by the addition of glucose (Fig. 4). In the previous study, gene knock-out or knock-down was employed to slow down the carbon flux of glycolysis and pentose phosphate pathways to enhance the supply of precursor G-6-P [18]. However, multiple knock-out/knock-down of key genes in glycolysis and pentose phosphate pathways severely inhibited the cell growth even when glycerol was used as the carbon source. In contrast, using the dynamic regulation strategy established in this study, the cell growth was totally not affected when glycerol was used as the carbon source (Fig. 4A). Using this strategy, high inositol production and yield were obtained in JQ08, and the accumulation of by-product ethanol was significantly reduced.

Environmental stresses, such as oxygen condition, have great influence on fermentation performance of microbial cell factory. It is reported that in fully aerobic conditions more glucose could be channeled to pentose phosphate pathway in *P. pastoris*, while increased glycolytic flux could be observed under the hypoxic conditions due to the upregulated expression of glycolytic genes like *gap* [31]. Therefore, it is reasonable to speculate that other genes controlled by the pGAP promoter would be upregulated in the hypoxic conditions as well. In our study, the promoter of native *IPS* gene which catalyzed the rate-limiting step for inositol biosynthesis was replaced with pGAP. Thus, the expression of native *IPS* gene could be enhanced under the low oxygen conditions. RT-qPCR was then performed to verify the expression of *IPS* in JQ08 under aerobic (DO: 30%) and hypoxic (DO: 15%) conditions. Results showed that the expression of *IPS* in JQ08 under hypoxic condition was significantly upregulated (about 3.1-fold increase) compared to that under the aerobic condition, which confirmed our speculation (Additional file 1: Fig. S3). This might be the reason that the inositol production of JQ08 was greatly increased under the low DO conditions in high-cell-density fermentation.

## Conclusions

In conclusion, the GRAS yeast *P. pastoris* was successfully engineered as a high-inositol producer in this study by combining overexpression of native and heterologous genes. An effective dynamic regulation strategy was established and applied to enhance the inositol production from glucose. Using the dynamic regulation strategy, 30.71 g/L inositol was produced in an optimized fed-batch high-cell-density fermentation, which is the highest concentration achieved in yeast cell factory to date. The strategy developed herein could be readily adapted for the production of other high-value biochemicals.

## Methods

### Strains and culture media

All strains used in this study are listed in Table 1. *E. coli* strain Trans10 (Transgen Biotech, Beijing, China) was used for DNA cloning and plasmid propagation. *P. pastoris* GS115 was employed as the parental strain for genetic engineering and inositol production. *E. coli* Trans10 was routinely grown in Luria-Bertani (LB) medium supplemented with 100 µg/mL ampicillin as required. The recipes of media used for *P. pastoris* strains cultivation and selection were as follows: YPD medium: 20 g/L tryptone, 10 g/L yeast extract, 20 g/L glucose. BMGY medium: 20 g/L tryptone, 10 g/L yeast extract, 100 mM KH_2_PO_4_/K_2_HPO_4_ buffer (pH 6.0), 13.4 g/L YNB, 0.4 ppm biotin, 2% (v/v) glycerol. BMMY medium: 20 g/L tryptone, 10 g/L yeast extract, 100 mM KH_2_PO_4_/K_2_HPO_4_ buffer (pH 6.0), 13.4 g/L YNB, 0.4 ppm biotin, 1% (v/v) methanol. 100 µg/mL zeocin was added into the medium when needed.

**Table 1:** Bacterial strains and plasmids used in this study

Strains/Plasmids	Relevant characteristic	Sources
Strains *E. coli*		
Trans10	*F*^*−*^ *mcrA*, Δ*(mrr-hsdRMS-mcrBC)*, *φ80*, *lacZ*Δ*M15*, Δ*lacX74*, *recA1*, *ara*Δ*139*, Δ*(ara-leu)7697*, *galU*, *galK*, *rpsL(Str*^*R*^*)*, *endA1*, *nupG*	Transgen Biotech
*P. pastoris*		
GS115	*his4* ^-^	Invitrogen
JQ01	Derived from GS115, the promoter of *IPS* gene was replaced with pGAP	This work
JQ02	Derived from JQ01, with *PpITR1* and *PpITR2* genes deleted	This work
JQ03	Derived from JQ02, with *pfk2* gene deleted	This work
JQ04	Derived from JQ03, overexpression of *ScIPS* gene from *S. cerevisiae* and *EcIMP* gene from *E. coli*	This work
JQ05	Derived from JQ04, the promoter of *zwf* gene was replaced with pGUT1	This work
JQ06	Derived from JQ04, the promoter of *pgi* gene was replaced with pGUT1	This work
JQ07	Derived from JQ04, the promoters of *pgi* and *zwf* genes were replaced with pGUT1	This work
JQ08	Derived from JQ07, the promoter of *pfk1* gene was replaced with pGUT1	This work
Plasmids		
pGAPZ A	ColE1 ori, Zeo^R^, pGAP	Invitrogen
pPIC9K	ColE1 ori, pAOX1 promoter, PpHIS4, Amp^R^, Kan^R^	Invitrogen
pEASY-T3	ColE1 ori, Amp^R^, TA cloning vector	Transgen Biotech
pJQ	pEASY-T3 derivative; Zeo^R^, pAOX-*mazF*	This work
pJQ01	pJQ derivative for markerless replacing the promoter of *IPS* gene with pGAP	This work
pJQ02-T1	pJQ derivative for markerless deletion of possible inositol transporter gene *PpITR1*	This work
pJQ02-T2	pJQ derivative for markerless deletion of possible inositol transporter gene *PpITR2*	This work
pJQ03	pJQ derivative for markerless deletion of *pfk2* gene	This work
pJQ04	pJQ derivative for markerless knock‑in of *ScIPS* and *EcIMP* expression cassettes in the *PpITR1* locus	This work
pJQ05	pJQ derivative for markerless replacing the promoter of *zwf* gene with pGUT1	This work
pJQ06	pJQ derivative for markerless replacing the promoter of *pgi* gene with pGUT1	This work
pJQ08	pJQ derivative for markerless replacing the promoter of *pfk1* gene with pGUT1	This work

### Plasmid construction

All primers and plasmids used in this study can be found in Additional file 1: Table S1 and Tables 1, respectively. To construct the markerless editing plasmid pJQ (Additional file 1: Fig. S2A), the toxic *mazF* gene and antibiotic zeocin resistance gene were used as the selection markers. Firstly, the *mazF* gene from *E. coli* was amplified and cloned into the *Bam*HI and *Age*I sites of pPIC9K. Then, the pAOX-*mazF* expression cassette was amplified from the obtained plasmid, fused with ZeoR expression cassette (from pGAPZ A plasmid) by overlapping PCR and cloned into pEASY-T3 vector (Transgen Biotech, Beijing, China), creating plasmid pJQ. All the gene editing plasmids used in this study were constructed based on the mother vector pJQ. For the construction of markerless gene deletion plasmid (Additional file 1: Fig. S2B), three homology arms, up-arm (~ 1000–1200 bp), short-arm (~ 100–300 bp), down-arm (~ 1000–1200 bp), were amplified from the genomic DNA of *P. pastoris* GS115 and inserted into the *Sac*II (up-arm) and *Spe*I (short-arm and down-arm) sites of pJQ vector, respectively. According to the genome editing demand, the sequence of short-arm was homologous to the 3’-end sequence of up-arm. For the construction of gene replacement or knock‑in plasmid, the cargo gene was inserted between the short-arm and down-arm of markerless gene deletion plasmid (Additional file 1: Fig. S2C). pGAP, pGUT1 and pHHX1 were amplified from the genomic DNA of *P. pastoris* GS115. *ScIPS* and *EcIMP* genes were obtained by PCR using the genomic DNA of *S. cerevisiae* and *E. coli*, respectively.

### Mutant screening

The pJQ derivative plasmids for gene deletion, replacement or knock‑in were first linearized by *Not*I and then transformed into *P. pastoris* by electroporation according to the published method [39]. The transformants were selected on YPD or BMGY plates containing 100 mg/L zeocin. Colonies appeared on the plates were then screened by colony PCR to detect the deletion of target genes and the insertion of *mazF*-*zeoR* counter-selection marker on the chromosome of *P. pastoris*. The positive colonies were inoculated into BMMY liquid medium to induce the expression of *mazF* gene. The obtained cultures were diluted and plated on the YPD or BMGY plates. Colony PCRs were subsequently performed to confirm the eviction of *mazF*-*zeoR* counter-selection marker and the insertion of cargo genes or promoters.

### Fed-batch fermentation

Shake-flask fermentations with various engineered strains were performed in 1 L shake flask with 200 mL reaction volume at 30 °C and 220 rpm. A modified inorganic salt medium (1.2 g/L KH_2_PO_4_, 0.5 g/L CaSO_4_, 6.5 g/L MgSO_4_, 5 g/L K_2_SO_4_, 18 g/L NH_4_H_2_PO_4_) containing 20 g/L glycerol was used as the fermentation medium. At 24 h of fermentation, 50 g/L glucose was supplemented into the medium for the inositol production. For the bioreactor fermentations, 10 L bioreactor (Shanghai Wanmuchun Biological Engineering Co., Shanghai, China) with a 7 L working volume was used. The initial glycerol concentration used in the inorganic salt medium was 50 g/L. After the glycerol was consumed, 50 g/L glucose was supplemented into the medium. Whenever the residual glucose concentration fell below 5 g/L, glucose solution (700 g/L) was pumped into the bioreactor to restore the glucose concentration to about 50 g/L. The temperature and pH were maintained at 30 °C and 5.0, respectively. The DO level was controlled at above 30% during 0–24 h (glycerol catabolism for cell growth) and then changed to 30%, 15% or 5% during the rest of fermentation period (glucose catabolism for inositol production) by automatic control of aeration rate and agitation speed. All *P. pastoris* strains were initially cultured in BMGY medium to prepare the seed culture (OD_600_ reached 10–12). Then, a 10% (v/v) inoculum of seed culture was used for all fed-batch fermentations. Shake-flask fermentations were performed in triplicate, while bioreactor fermentations were carried out in duplicate.

### Analytical methods

Fermentation samples were collected every 6 or 12 h for the analysis. Glucose, glycerol, inositol, and ethanol in the fermentation broth were determined using high-performance liquid chromatography system (LC-20 A, Shimadzu, Kyoto, Japan) equipped with Agilent Hi-Plex Ca column (Agilent Technologies, Santa Clara, CA, USA) or Waters Sugar-Pak I column (Waters, Milford, MA, USA) and refractive index detector (RID). The mobile phase was ddH_2_O at a flow rate of 0.6 mL/min at 80 °C. Specifically, all the samples were filtered through the 0.22 μm filter before analysis. Cell density was monitored using a microplate reader at 600 nm (OD_600_). WCW was measured by centrifuging 1 mL of fermentation broth in a previously weighed 1.5 mL centrifuge tube at 12,000*g* for 2 min. The supernatant was discarded, and the difference in weight was defined as the WCW.

### RT-qPCR analysis

The primers used in RT-qPCR are shown in Additional file 1: Table S1. *P. pastoris* strains were cultivated in inorganic salt medium with 20 g/L glucose or glycerol as carbon source. When OD_600_ reached about 10 (exponential growth phase), cells were harvested by centrifugation at 12,000*g* for 2 min. Total RNA extraction (RNA-easy Isolation Reagent, Vazyme, Beijing, China) and reverse transcription of cDNA (HiScript III RT SuperMix for qPCR, Vazyme, Beijing, China) were performed following the manufacturer’s instructions. All qPCRs were conducted according to the published procedure by using the ChamQ Universal SYBR qPCR Master Mix (Vazyme, Beijing, China) and the QuantStudio 6 Flex system (Applied Biosystems, Life Technologies, CA, USA) [40]. 2^−∆∆Ct^ method was used to evaluate the transcription levels of the target genes [41], where the *arg4* gene was used as the internal standard. All samples were run in triplicate.

## Supplementary Information

**Additional file** **1:** **Table S1.** Primers used in this study. **Fig. S1. **Comparation of genomeediting tool developed by Yang et al. [1] or used in ourstudy. A CYC1TT scar would leave in the genome after each round of gene editing in theprevious method. However, markerless genome editing could be achieved using ourmethod. **Fig. S2.** Maps of plasmids usedfor markerless genome editing in *P. pastoris*. **A** Plasmid pJQ was usedas the mother vector for the construction of markerless gene deletion orknock-in plasmids. **B** Map of plasmid used for markerless gene deletion. **C**Map of plasmid used for markerless gene knock-in. **Fig.S3.** qPCR results showing the mRNA expressions of *IPS*gene of JQ08 under aerobic (DO: 30%) and hypoxic (DO: 15%) conditions.

## Data Availability

All data generated or analyzed during this study are included in this published article and its additional information file.
