# Supplementary material for: Metabolic engineering of Pichia pastoris for myo-inositol production by dynamic regulation of central metabolism
Source: Microb Cell Fact. 2022 Jun 3;21:112. doi: 10.1186/s12934-022-01837-x (PMC9166411; doi:10.1186/s12934-022-01837-x)
Supplement: Supplementary file 1 — Additional file 1: Table S1. Primers used in this study. Fig. S1. Comparation of genomeediting tool developed by Yang et al. [1] or used in ourstudy. A CYC1TT scar would leave in the genome after each round of gene editing in theprevious method. However, markerless genome editing could be achieved using ourmethod. Fig. S2. Maps of plasmids usedfor markerless genome editing in P. pastoris. A Plasmid pJQ was usedas the mother vector for the construction of markerless gene deletion orknock-in plasmids. B Map of plasmid used for markerless gene deletion. CMap of plasmid used for markerless gene knock-in. Fig.S3. qPCR results showing the mRNA expressions of IPSgene of JQ08 under aerobic (DO: 30%) and hypoxic (DO: 15%) conditions. [file 12934_2022_1837_MOESM1_ESM.docx]

# Additional file

**Metabolic engineering of** ***Pichia pastoris* for** ***myo*-inositol production by dynamic** **regulation of central metabolism**

Qiquan Zhang#, Xiaolu Wang#, Huiying Luo, Yaru Wang, Yuan Wang, Tao Tu, Xing Qin, Xiaoyun Su, Huoqing Huang, Bin Yao, Yingguo Bai*, Jie Zhang*

State Key Laboratory of Animal Nutrition, Institute of Animal Science, Chinese Academy of Agricultural Sciences, Beijing 100193, China

# Qiquan Zhang and Xiaolu Wang contributed equally to this work.

* Corresponding author:

Yingguo Bai

No.2 Yuanmingyuan West Road, Haidian district

Beijing, 100193 China

Tel: +86-10-62599910

E-mail: baiyingguo@caas.cn

Jie Zhang

No.2 Yuanmingyuan West Road, Haidian district

Beijing, 100193 China

Tel: +86-10-62599910

E-mail: [zhangjie09@caas.cn](mailto:zhangjie09@caas.cn)

**Table S1** Primers used in this study

| Primers (pair) | Sequences |
| --- | --- |
| Construction of plasmid pJQ | |
| *mazF* gene | 5'-GATCAAAAAACAACTAATTATTCGAAATGGTAAGCCGATACGTACC-3'  5'-CCTCTTGATTAGAATCTAGCAAGCTACCCAATCAGTACG-3' |
| pAOX-*mazF* | 5'-GATCTAACATCCAAAGACGAAAGGTTGAATGAAACCTTTTTGCC-3'  5'-gaagctatggtgtgtgggTCTCACTTAATCTTCTGTAC-3' |
| Zeocin gene | 5'-GAAGATTAAGTGAGAcccacacaccatagcttcaaaatg-3'  5'-gcaaattaaagccttcgagcgtcccaaaacc-3' |
| Enhancement of inositol biosynthetic pathway | |
| pGAP promoter | 5'-CAATTTCGATATAAATATATGTTTTTTGTAGAAATGTCTTGGTGTCCTCGTCCAATC-3'  5'-GGAGTGTATTGAATAGTCATTGTGTTTTGATAGTTGTTCAATTGATTG-3' |
| Homology arms  (for replacing the promoter of *IPS* gene with pGAP) | 5'-gtcgaAGGGCGATCCCAATCATTATTTTGCCGGGGC-3'  5'-CCAGACCTTTAGTTATCCTTCGAACAGCTATAATAGCAATGTCTTCTTC-3'  5'-ggctttaatttgcCGAAGGATAACTAAAGGTCTGGTC-3'  5'-CACCAAGACATTTCTACAAAAAACATATATTTATATCGAAATTGGAACC-3'  5'-GAACAACTATCAAAACACAATGACTATTCAATACACTCCTAAAGTCC-3'  5'-CCGCGAATTCACTAGCCAAGAATTGTGCCAGCACAGAC-3' |
| *IPS* gene from *S. cerevisiae* | 5'-CTTGTGTCTATCGTAGTAAAAATGACAGAAGATAATATTGCTCCAATCACCTCCG-3'  5'-CGACCAAGAGACGCCCGTTACAACAATCTCTCTTCGAATCTTAG-3' |
| *IMP* gene from *E. coli* | 5'-GAATAAAGAGAAATTTTATTTAACGCTTCAGAGCGTCGCTTAACTCGTCACGCATG-3'  5'-CAAATAATCAATAAATATGCATCCGATGCTGAACATCGCCGTGCGC-3' |
| Homology arms  (for markerless knock‑in *ScIPS* and *EcIMP* expression cassettes) | 5'-CGCCATGGCGGCCGCAAAATGTCTGGTTCTAATTTC-3'  5'-CAAGGGCGATCCAATCGAATTCCCGCTGGTAGTTTTTAGTAAACTAG-3'  5'-GGCGATCCCAATCAGTACCCCTTGTGGGTCTC-3'  5'-CGGAAACAGTGCCAATCGAACGCAGCTGGTAGTTTTTAGTAAAC-3'  5'-GAGATTGTTGTAACGGGCGTCTCTTGGTCGAACGCC-3'  5'-GCAGGCGGCCGCGAATTCACTAGTGTTGATTTATTTGCAATATCTTG-3'  5'-CGCCATGGCGGCCGCAAAATGTCTGGTTCTAATTTC-3'  5'-CGCCATGGCGGCCGCAAAATGTCTGGTTCTAATTTC-3' |
| *PpITR1*, *PpITR2* and *pfk2* deletion | |
| Homology arms  (for *PpITR1* deletion) | 5'-CGCCATGGCGGCCGCAAAATGTCTGGTTCTAATTTC-3'  5'-CAAGGGCGATCCAATCGAATTCCCGCTGGTAGTTTTTAGTAAACTAG-3'  5'-GGCGATCCCAATCAGTACCCCTTGTGGGTCTC-3'  5'-CGACCAAGAGACGCCCGGCTGGTAGTTTTTAGTAAAC-3'  5'-GTTTACTAAAAACTACCAGCCGGGCGTCTCTTGGTCGAACG-3'  5'-GCAGGCGGCCGCGAATTCACTAGTGTTGATTTATTTGCAATATCTTG-3' |
| Homology arms  (for *PpITR2* deletion) | 5'-GCCATGGCGGCCGCAAAGAGATACAAGCTAAACAAG-3'  5'-CAAGGGCGATCCAATCGAATTCCCGTCTATAAAACTTTAATAGAGAGGG-3'  5'-gctAGGGCGATCCCAATCAATACAATTCAGAGCAGACTTGGC-3'  5'-GAAATTACGATAGTTGACAACAAAGTCTATAAAACTTTAATAGAGAGGGTAG-3'  5'-CTCTCTATTAAAGTTTTATAGACTTTGTTGTCAACTATCGTAATTTCATTAAACTG-3'  5'-GGCCGCGAATTCACTAGCGTTTAGTTTCC-3' |
| Homology arms  (for *pfk2* deletion) | 5'-cggccgcggattacggctccctc-3'  5'-cgaattcccgcgggatgcaggtttctctggg-3'  5'-ggcgatcccaatcactagttcatcaacagatgccacgatcgg-3'  5'-cagtatgttccttatgtaagaatgcgatgcaggtttctctgggatcaag-3'  5'-cccagagaaacctgcatcgcattcttacataaggaacatactgaaagtatgg-3'  5'-ggcggccgcgaattcactagtaatcttgtactttgaaagtggg-3' |
| *pgi*, *zwf* and *pfk1* regulation | |
| pGUT1 promoter  (for *pgi* regulation) | 5'-GCCCGTTCGCAGAAAAAACTTGAGATGCATGGACGGAATCAAAC-3'  5'-CCTCTTGCAATAGAGACGGCATTATAGTAGATATATCTGTGGTATAGTGTG-3' |
| Homology arms  (for *pgi* regulation) | 5'-CGCCATGGCGGCCGCTGAAAGTAATTGAAGTGG-3'  5'-GCGATCCAATCGAATTCCCTTTTTCTGCGAACGGGCAAGTG-3'  5'-ctAGGGCGATCCCAATCAATTCAGCAGTCTAGGCATCAAGACG-3'  5'-CGTCCATGCATCTCAAGTTTTTTCTGCGAACGGGCAAGTGTG-3'  5'-CACAGATATATCTACTATAATGCCGTCTCTATTGCAAGAGGACAATGCTAC-3'  5'-CGGCCGCGAATTCACTAGCACCAATAACTGGGATGTTTTGC-3' |
| pGUT1 promoter  (for *zwf* regulation) | 5'-GACACCATGGGATATCCCCTGATTACTTGAGATGCATGGACGGAATCAAACACGG-3'  5'-TTCTACGGCTTTCGTATCGGTCATTATAGTAGATATATCTGTGGTATAGTG-3' |
| Homology arms  (for *zwf* regulation) | 5'-TCCCGGCCGCCATGGCGGCCGCGGCAAGGAATCTACTTGGGAGTT-3'  5'-GTTAGATCAAGGGCGATCCAATCGAATTCAATCAGGGGATATCCCATGGTGTCAACCTAGAGA-3'  5'-tttgcaagctAGGGCGATCCCAATCCGTTAGCCAATAGTGTCCCTGCATTCTGGTTC-3'  5'-CCGTGTTTGATTCCGTCCATGCATCTCAAGTAATCAGGGGATATCCCATGGTGTC-3'  5'-TATACCACAGATATATCTACTATAATGACCGATACGAAAGCCGTAGAATTTGTGGGCC-3'  5'-AGGCGGCCGCGAATTCACTAGTCTTCCCACCTTTCTGTGTTGATGTGCAAGCC-3' |
| pGUT1 promoter  (for *pfk1* regulation) | 5'-GTGATTTTCTTAGTCTCGAAGACTTGAGATGCATGGACGGAA-3'  5'-GCACTTATAGATGGTTCTGGCATTATAGTAGATATATCTGTGGTA-3' |
| Homology arms  (for *pfk1* regulation) | 5'-cggccgccatggcggccgcggTGGAACTGCGAGTTCTTCGA-3'  5'-caagggcgatccaatcgaattcCTTCGAGACTAAGAAAATCACCT-3'  5'-tttgcaagctagggcgatcccaatcACTACAACTTTTAGCTTTTAG-3'  5'-GATTCCGTCCATGCATCTCAAGTCTTCGAGACTAAGAAAATCACCT-3'  5'-CTATACCACAGATATATCTACTATAATGCCAGAACCATCTATAAGT-3'  5'-aggcggccgcgaattcactagtGTTCCTTTATCAGTTCAGGCC-3' |
| RT-qPCR | |
| *arg4* | 5'-GACGAACTAAGTGAGATTCATCGTGG-3'  5'-CCTTACCAGAGATTCCACGACCAA-3' |
| *zwf* | 5'-GGAGAGACCCGTCTCTAATG-3'  5'-CATCCACATAAGCTGGCTTC-3' |
| *pgi* | 5'-CATGGTGACAGAGGCATTG-3'  5'-CGTTACGGATGGTTTCAGC-3' |
| *pfk1* | 5'-CGTCAGCTGGTAGAGAGTTG-3'  5'-CTTCACCGGGCAAGTCTTTG-3' |
| *IPS* | 5'-CTGTCTACTACCCAGATTTCA-3'  5'-CGGCAATGTCACTTCTAA-3' |


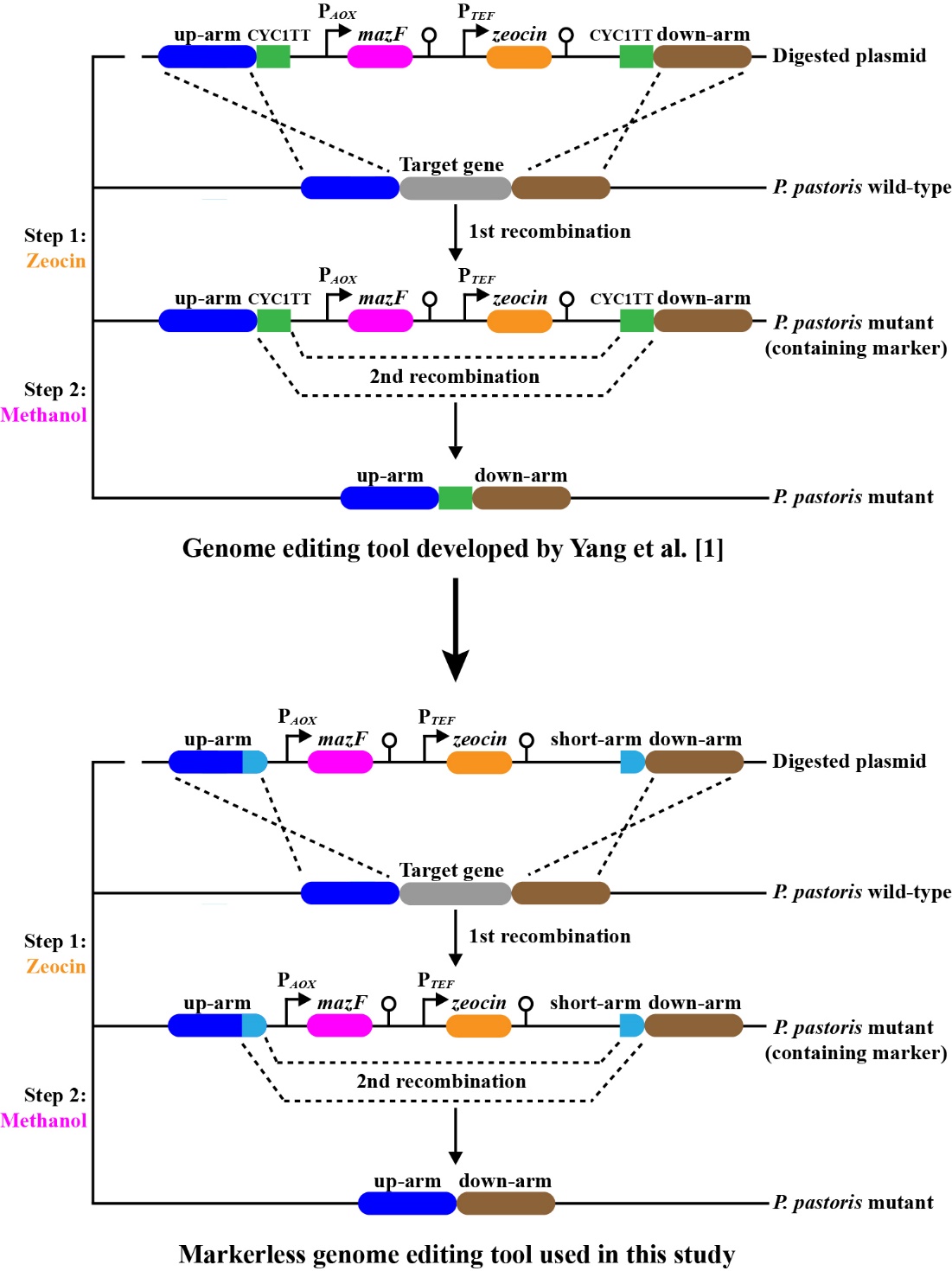


**Fig. S1** Comparation of genome editing tool developed by Yang et al. [[1](#_ENREF_1)] or used in our study. A CYC1TT scar would leave in the genome after each round of gene editing in the previous method. However, markerless genome editing could be achieved using our method.


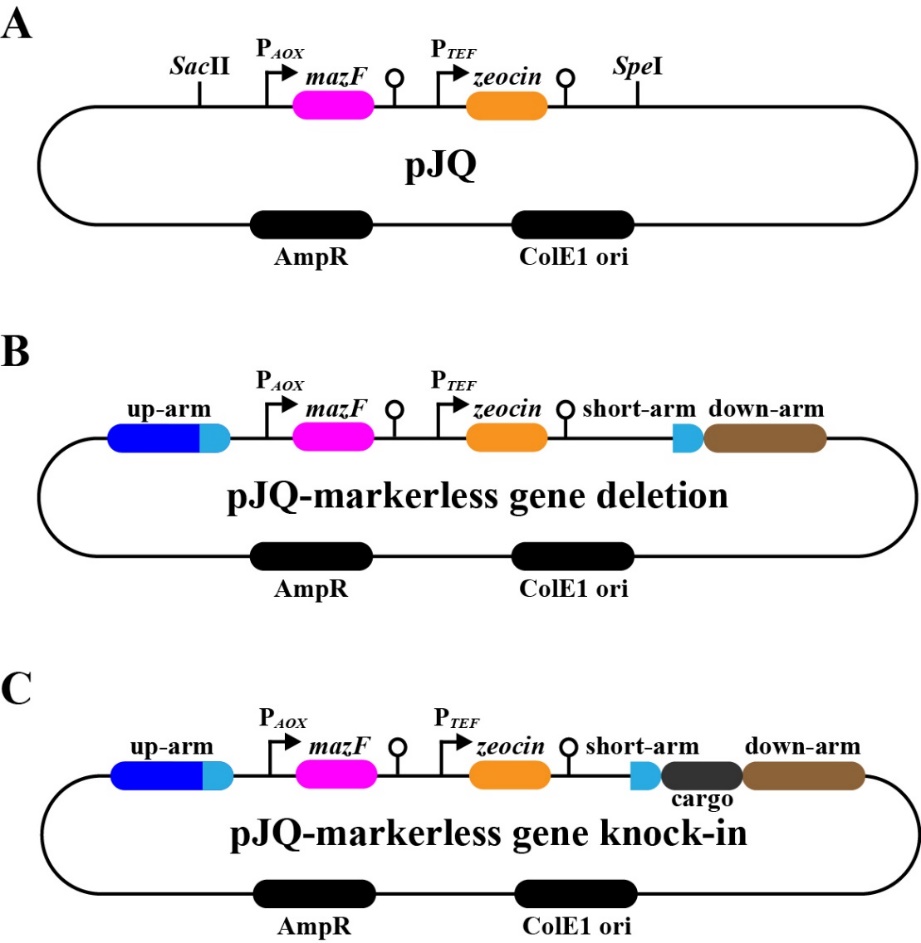


**Fig. S2** Maps of plasmids used for markerless genome editing in *P. pastoris*. **A** Plasmid pJQ was used as the mother vector for the construction of markerless gene deletion or knock-in plasmids. **B** Map of plasmid used for markerless gene deletion. **C** Map of plasmid used for markerless gene knock-in.


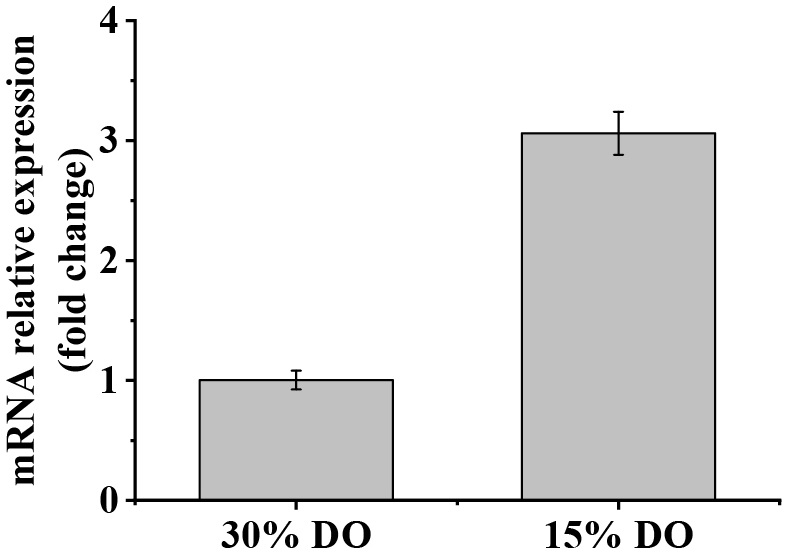


**Fig. S3** qPCR results showing the mRNA expressions of *IPS* gene of JQ08 under aerobic (DO: 30%) and hypoxic (DO: 15%) conditions.

**Reference**

1. Yang J, Jiang W, Yang S. *mazF* as a counter-selectable marker for unmarked genetic modification of *Pichia pastoris*. FEMS Yeast Res. 2009;9:600-9.
